# Supplementary material for: Purine nucleotide depletion prompts cell migration by stimulating the serine synthesis pathway
Source: Nat Commun. 2022 May 16;13:2698. doi: 10.1038/s41467-022-30362-z (PMC9110385; doi:10.1038/s41467-022-30362-z)
Supplement: Supplementary file 1 — Supplementary Information [file 41467_2022_30362_MOESM1_ESM.pdf]

Supplementary Information for

Purine nucleotide depletion prompts cell migration by stimulating the serine synthesis pathway

Mona Hoseini Soflaee[1,†], Rushendhiran Kesavan[1,†], Umakant Sahu[2,3†], Alpaslan Tasdogan[4], Elodie Villa[2,3], Zied Djabari[2,3], Feng Cai[1], Diem H. Tran[1], Hieu S. Vu[1], Eunus S. Ali[2,3], Halie Rion[1], Brendan P. O'Hara[2,3], Sherwin Kelekar[1], James Hughes Hallett[5], Misty Martin[1], Thomas P. Mathews[1], Peng Gao[6], John M. Asara[7], Brendan D. Manning[5,*], Issam Ben-Sahra[2,3*], Gerta Hoxhaj[1,*]

*Correspondence to: bmanning@hsph.harvard.edu; issam.ben-sahra@northwestern.edu; gerta.hoxhaj@utsouthwestern.edu.

[†]These authors contributed equally to this work.

This PDF file includes:

Supplementary Figures 1-6

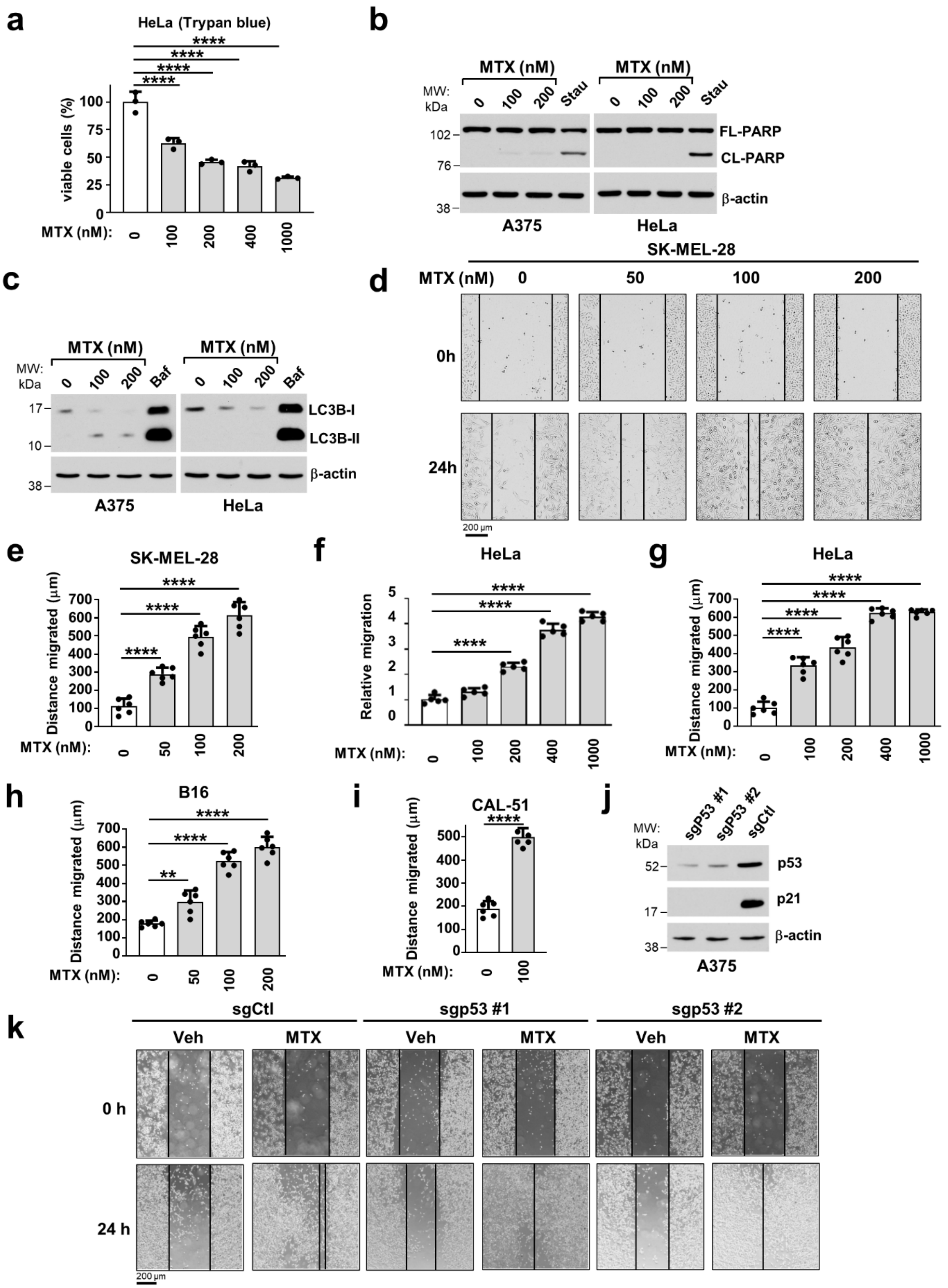

a
HeLa (Trypan blue)
viable cells (%)
MTX (nM):
b
MTX (nM)
MW: kDa
Stau
FL-PARP
CL-PARP
β-actin
A375
HeLa
c
Baf
LC3B-I
LC3B-II
d
SK-MEL-28
0h
24h
200 µm
e
SK-MEL-28
Distance migrated (µm)
f
HeLa
Relative migration
g
HeLa
h
B16
i
CAL-51
j
sgP53 #1
sgP53 #2
sgCtl
p53
p21
k
sgCtl
sgp53 #1
sgp53 #2
Veh
MTX
0 h
24 h

Supplementary Figure 1: Purine depletion does not induce cell death and supports cell migration independently of the p53 status.

a, Relative cell viability (Trypan blue) of HeLa cells treated with either vehicle (DMSO) or with the indicated doses of MTX for 72 h. **b,** Immunoblots of A375 and HeLa cells treated with either vehicle (DMSO), the indicated doses of MTX for 24 h, or staurosporine (Stau; 50 nM, 15 h). **c,** Immunoblots of A375 and HeLa cells treated with either vehicle (DMSO), the indicated doses of MTX for 24 h, or Bafilomycin A1 (Baf; 500 nM, 15 h). **d,** Representative images from a wound-healing assay after treatment with the indicated doses of MTX (24 h) in SK-MEL-28. **e,** Quantification of the migrated distance from a wound-healing assay (24 h) of experiment in (d). **f**, Quantification of the relative migration from a transwell assay in HeLa cells treated with vehicle (DMSO) or with the indicated doses of MTX for 24 h as in Fig. 1d. **g-i,** Quantification of the migrated distance from a wound-healing assay (24 h) in response to treatment with the indicated doses of MTX as in (Fig.1e), but from HeLa (g), B16 (h), and CAL-51 (i). **j,** Immunoblots of A375 cells subjected to control or two distinct guide RNA against the p53 gene. **k**, Representative images from a wound-healing assay using A375 cells treated as in (j). (a, e-i) Data are presented as the mean ± s.d from n=3 (a) or n=5 (f), n=6 (e,g-i) of biologically independent samples. (a, e-h) One-way ANOVA, Turkey's post-hoc test, multiple comparison, (i) Unpaired t-test, ***$p<0.001$, ****$p<0.0001$. Scale bars are indicated in the figures (d,k). Data are representative of n=2 (b,c,j,k) or n=3 (a,d-i) independent experiments. Source data and exact p values are provided as a Source Data file.

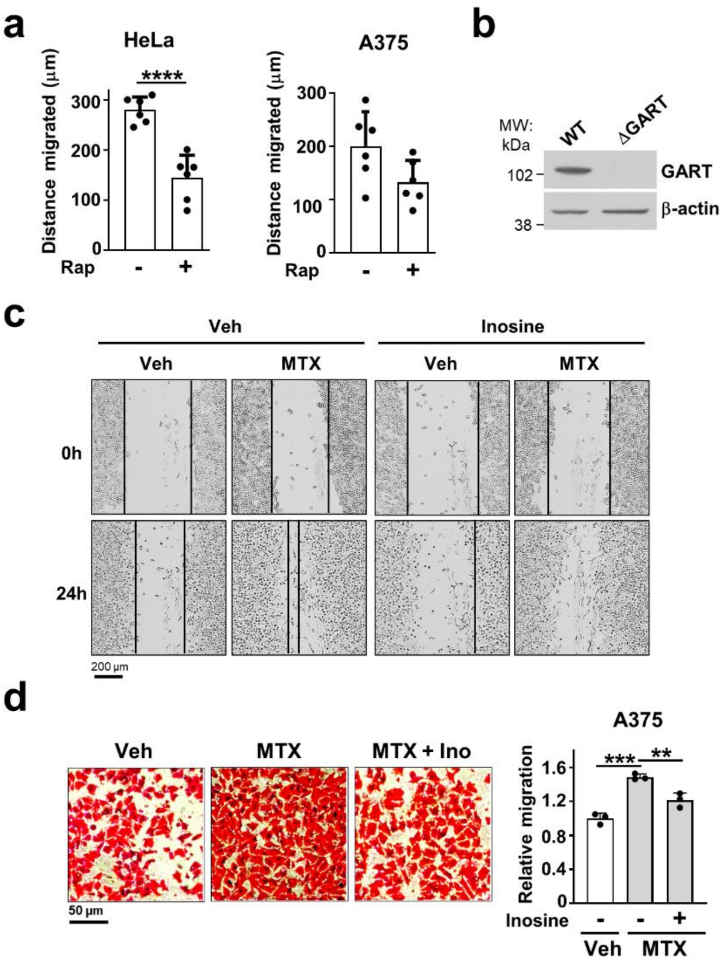

Supplementary Figure 2: Exogenous inosine averts the methotrexate-dependent stimulation of cell migration.

a, Quantification of the migrated distance as in Fig.1e from a wound-healing assay of HeLa or A375 cells treated with vehicle (Veh, DMSO) or rapamycin (20 nM) for 24 h. **b**, Immunoblots of wild-type or GART knockout (*ΔGART*) HeLa cells blotted with the indicated antibodies. Supporting Fig. 1h. **c,** Representative images from a wound-healing assay of Fig. 1i from A375 cells treated with vehicle (DMSO) or MTX (100 nM) for 24 h in the presence or absence of inosine (50µM). **d,** Representative images of A375 cells after migration through a transwell which were treated with vehicle (DMSO) or MTX (200 nM) for 24 h ins the presence or absence of inosine (50 µM). Quantification is shown from three independent biological replicates and presented as relative migration normalized to control (untreated). (a, d) Data are presented as the mean ± s.d from n=3 (d) or n=6 (a) of biologically independent samples. (a) Unpaired t-test, (d) One-way ANOVA, Turkey's post-hoc test, multiple comparison, *p<0.05, ****p<0.0001. (c, d) Scale bars are indicated in the figures. Data are representative of n=2 (b,d) or n=3 (a,c) independent experiments. Source data and exact p values are provided as a Source Data file.

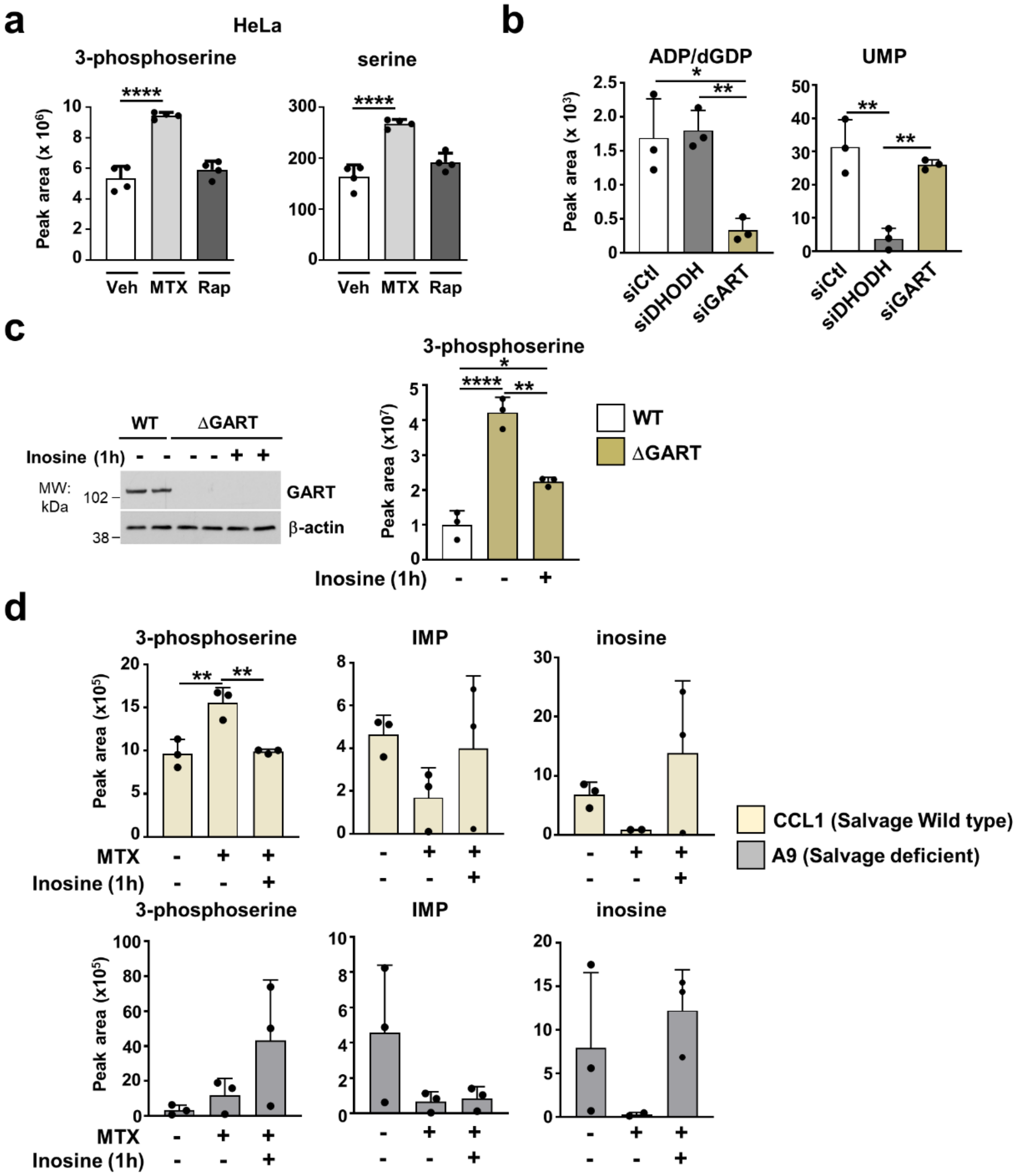

Supplementary Figure 3: De novo purine synthesis is required to maintain steady-state levels of 3-phosphoserine and serine.

a, Normalized peak areas of 3-phosphoserine and serine from HeLa cells treated with vehicle (Veh, DMSO), MTX (2 µM), or mTORC1 inhibitor rapamycin (Rap, 20 nM) for 8 h and measured with LC-MS/MS. **b,** Normalized peak areas of the indicated metabolites measured by LC-MS/MS from HeLa cells transfected with either nontargeting siRNA controls (siCtl), or siRNA against DHODH or GART (in support of Fig. 2b). **c,** Immunoblots and normalized peak areas of 3-phosphoserine measured by LC-MS/MS from wild-type or *ΔGART* HeLa cells cultured either without inosine for 15 h or treated with inosine (50 µM) for the last hour prior to metabolite extraction. **d,** Normalized peak areas of the indicated metabolites from wild-type (CCL1) or purine salvage-deficient APRT and HPRT knockout fibroblasts (A9) that were treated with MTX (2 µM)

for 15h followed by inosine (50 μM) addition in the last hour. (a-d) Data are presented as the mean ± s.d from n=3 (b-d) or n=4 (a) of biologically independent samples. (a-d) One-way ANOVA, Turkey's post-hoc test, multiple comparison, *p<0.05, **p<0.01, ***p<0.001, ****p<0.0001. Data are representative of n=2 (b-d) or n=3 (a) independent experiments. Source data and exact p values are provided as a Source Data file.

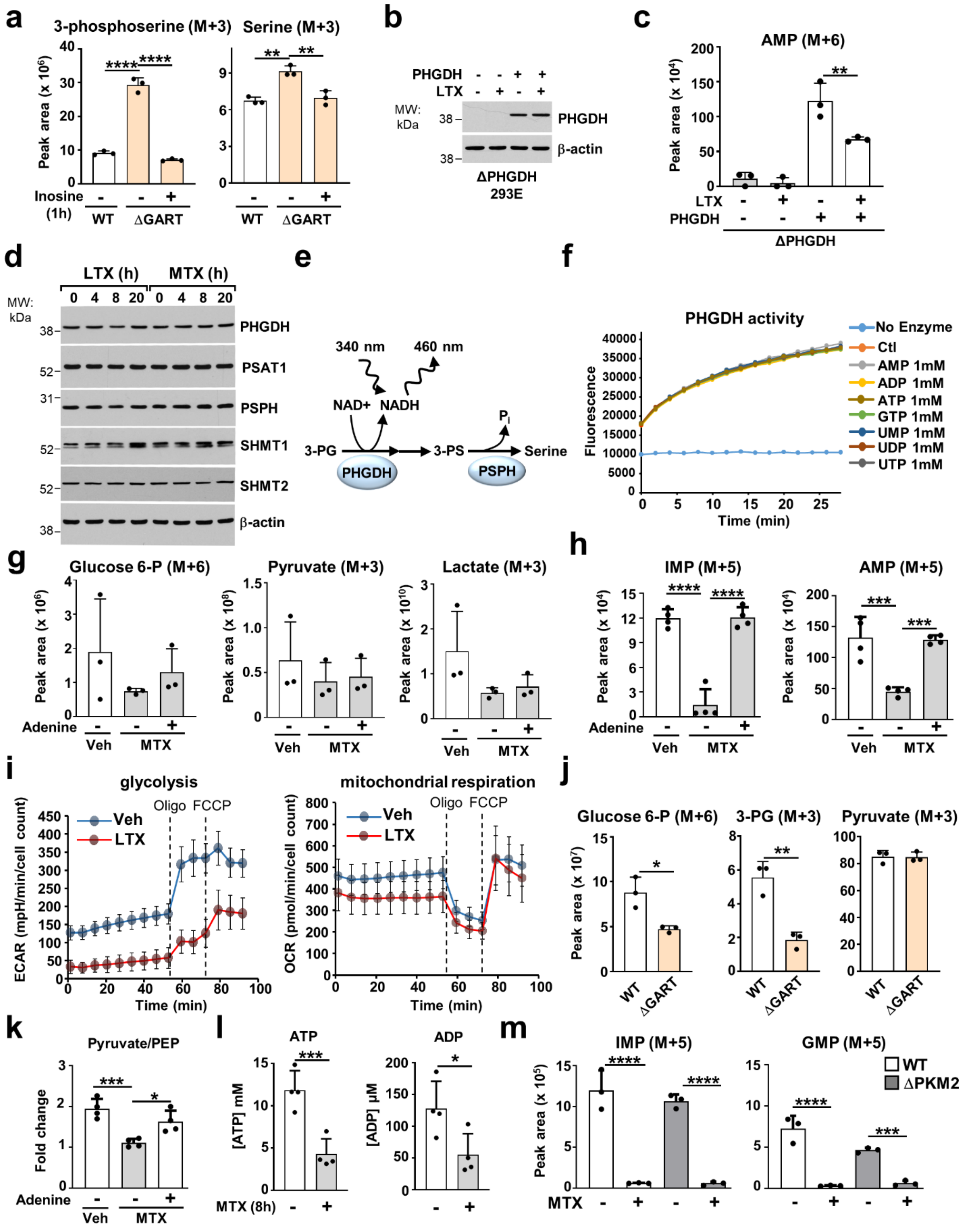
a
3-phosphoserine (M+3)
Serine (M+3)
Peak area (x 10⁶)
Inosine (1h)
WT
ΔGART
b
PHGDH
LTX
MW: kDa
β-actin
ΔPHGDH 293E
c
AMP (M+6)
Peak area (x 10⁴)
LTX
PHGDH
ΔPHGDH
d
LTX (h)
MTX (h)
PHGDH
PSAT1
PSPH
SHMT1
SHMT2
β-actin
e
340 nm
460 nm
NAD+
NADH
3-PG
3-PS
Serine
PHGDH
PSPH
f
PHGDH activity
Fluorescence
Time (min)
No Enzyme
Ctl
AMP 1mM
ADP 1mM
ATP 1mM
GTP 1mM
UMP 1mM
UDP 1mM
UTP 1mM
g
Glucose 6-P (M+6)
Pyruvate (M+3)
Lactate (M+3)
Adenine
Veh
MTX
h
IMP (M+5)
AMP (M+5)
i
glycolysis
mitochondrial respiration
Oligo
FCCP
Veh
LTX
ECAR (mpH/min/cell count)
OCR (pmol/min/cell count)
Time (min)
j
Glucose 6-P (M+6)
3-PG (M+3)
Pyruvate (M+3)
Peak area (x 10⁷)
k
Pyruvate/PEP
Fold change
l
ATP
ADP
[ATP] mM
[ADP] μM
MTX (8h)
m
IMP (M+5)
GMP (M+5)
WT
ΔPKM2
Peak area (x 10⁵)
MTX

Supplementary Figure 4: Purine levels control the metabolic flux through de novo serine synthesis.

a, Normalized peak areas of 3-phosphoserine (3-PS) (M+3) and serine (M+3) from wild-type or (*ΔGART*) HeLa cells grown in the absence of nucleotides and stimulated with inosine (50 µM) and labelled with $^{13}C_6$-glucose in the final hour. **b**, Immunoblots of HEK293E cells cultured in 10% dialyzed serum validating the CRISPR-mediated PHGDH knockout, and treated with vehicle (DMSO) or LTX (2 µM) for 8 h. **c**, Normalized peak areas of de novo synthesized AMP from *ΔPHGDH* HEK293E cells reconstituted or not with cDNA expressing *PHGDH*, that were treated with either vehicle (Veh, DMSO) or LTX (2 µM) for 8 h, and labelled with $^{13}C_6$-glucose in the last hour. (Related to Fig. 3d). **d,** Immunoblots of HeLa cells cultured in 10% dialyzed serum and treated with vehicle (DMSO) or MTX (2 µM) or LTX (2 µM) at the indicated times. Levels of metabolic enzymes of the serine metabolism are evaluated. **e,** Schematic illustrating the PHGDH enzymatic assay. **f,** PHGDH enzymatic assay was measured in the presence or absence of the indicated nucleotides (1 mM) (See methods). The data is representative of three independent experiments. **g,** Normalized peak areas of the glycolytic intermediates from HeLa cells that were treated with either vehicle (Veh, DMSO) or MTX (2 µM) for 15 h, followed by 1 h of adenine supplementation (50µM, 1 h), and labelled with $^{13}C_6$-glucose in the last hour. **h,** Normalized peak areas of the indicated metabolites from that were treated with either vehicle (Veh, DMSO) or MTX (2 µM) for 15 h, followed by 1 h of adenine supplementation (50µM, 1 h), and labelled with $^{13}C_6$-glucose in the last hour. (Related to Fig. 3b). **i,** Measurement of the extracellular acidification rate (ECAR) and oxygen consumption rate (OCR) from HeLa cells treated with vehicle (DMSO) or LTX (2 µM) for 15 h. **j,** Normalized peak areas of the indicated metabolites from wild-type or (*ΔGART*) HeLa cells grown in the absence of nucleotides for 24 h. **k,** Ratio of Pyruvate/PEP from steady-state metabolomics of HeLa cells that were treated with either vehicle (Veh, DMSO) or MTX (2 µM) for 15 h, followed by 1 h of adenine supplementation (50 µM, 1 h). **l,** Intracellular concentration of ADP and ATP are shown for HeLa cells treated with vehicle (Veh) or MTX (2 µM, 8 h). Absolute concentrations were calculated by LC–MS/MS using internal standards for ADP and ATP (See methods). **m,** Normalized peak areas of the indicated metabolites from wild-type and knockout PKM2 (*ΔPKM2*) MEFs, treated with vehicle (Veh, DMSO) or MTX (2 µM) for 8h, and labeled for the last 3h with $^{13}C_6$-glucose (in support of Fig. 3j). (a,c,g-m) Data are presented as the mean ± s.d n=3 (a,c,g,j,m), or n= 4 (h,k,l), or n=6 (i) of biologically independent samples. (a,c,g,h,k,m) One-way ANOVA, Turkey's post-hoc test, multiple comparison, (j, l) Unpaired t-test, *p<0.05, **p<0.01, ***p<0.001, ****p<0.0001. Data are representative of n=2 (b-d,f,i-l) or n=3 (a,g,h,m) independent experiments. Source data and exact p values are provided as a Source Data file.

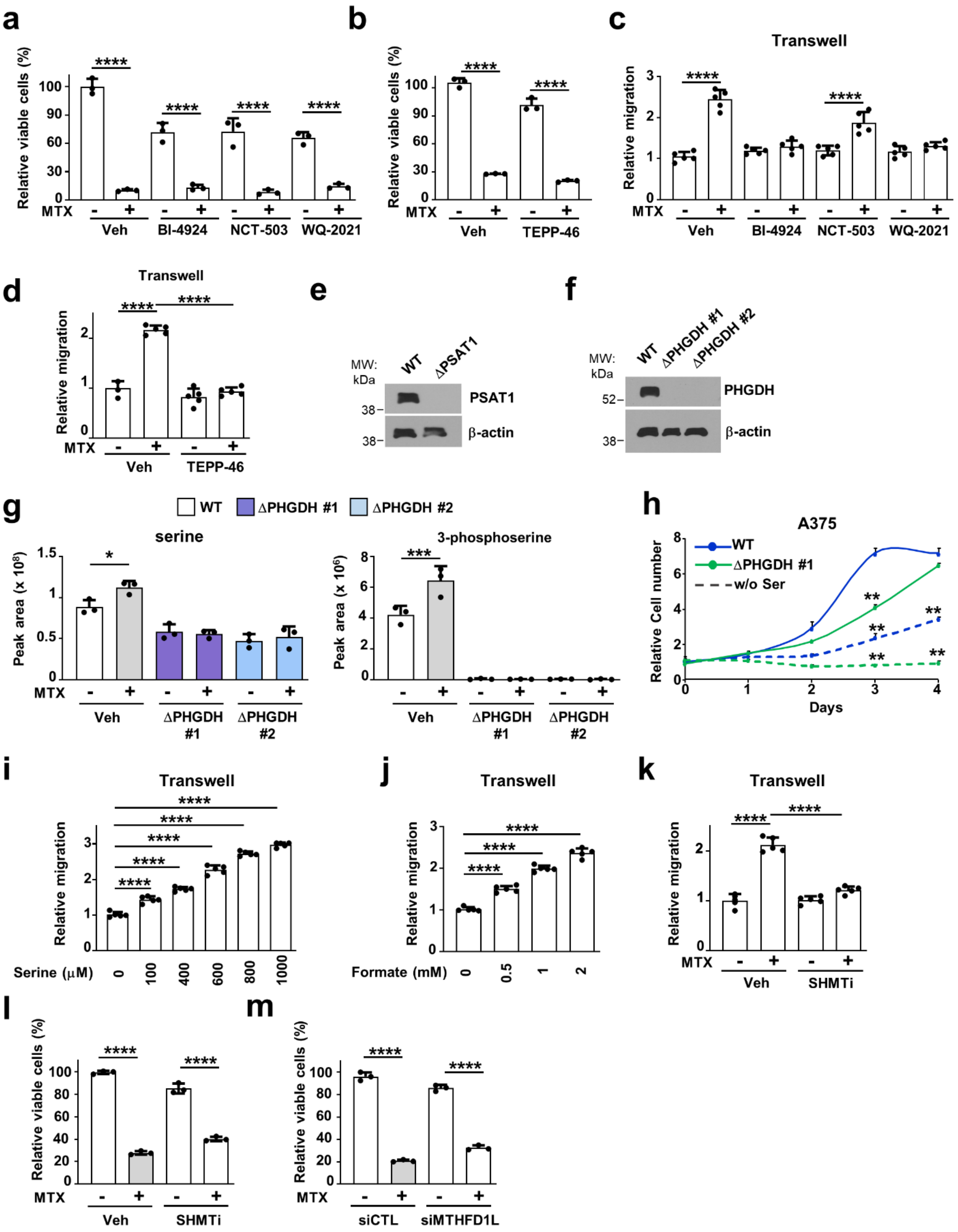
a
Relative viable cells (%)
MTX
Veh
BI-4924
NCT-503
WQ-2021
b
Relative viable cells (%)
MTX
Veh
TEPP-46
c
Transwell
Relative migration
MTX
Veh
BI-4924
NCT-503
WQ-2021
d
Transwell
Relative migration
MTX
Veh
TEPP-46
e
MW: kDa
WT
ΔPSAT1
PSAT1
β-actin
f
MW: kDa
WT
ΔPHGDH #1
ΔPHGDH #2
PHGDH
β-actin
g
WT
ΔPHGDH #1
ΔPHGDH #2
serine
Peak area (x 10[8])
MTX
Veh
ΔPHGDH #1
ΔPHGDH #2
3-phosphoserine
Peak area (x 10[6])
h
A375
WT
ΔPHGDH #1
w/o Ser
Relative Cell number
Days
i
Transwell
Relative migration
Serine (μM)
j
Transwell
Relative migration
Formate (mM)
k
Transwell
Relative migration
MTX
Veh
SHMTi
l
Relative viable cells (%)
MTX
Veh
SHMTi
m
Relative viable cells (%)
MTX
siCTL
siMTHFD1L

Supplementary Figure 5: Serine and formate levels support the methotrexate-dependent induction in cell migration.

a, Relative viability (Trypan blue) of A375 cells treated with either vehicle (Veh, DMSO) or MTX (100 nM) for 48 h in the presence or absence of two distinct selective PHGDH inhibitors (BI-4924, 15 µM; NCT-503, 10 µM, WQ-2021, 50 µM). **b,** Relative viability (Trypan blue) of A375 cells treated with either vehicle (Veh, DMSO) or MTX (100 nM) for 48 h in the presence or absence of TEPP-46 (100 µM). **c,** Quantification of the relative cell migration (transwell assay, 12 h) of A375 cells after MTX treatment (100 nM) in the presence or absence of three distinct PHGDH inhibitors (BI-4924, 15 µM and NCT503, 10 µM, WQ-2021, 50 µM). **d,** Quantification of the relative cell migration (transwell assay, 12 h) of A375 cells after MTX treatment (100nM) in the presence or absence of TEPP-46 (100 µM). **e,** Immunoblots of PSAT1 from Hela wild-type or PSAT1 knockout (*ΔPSAT1*). **f,** Immunoblots of PHGDH from A375 wild-type or PHGDH knockout (*ΔPHGDH*) from two independent knockout clones (#1, #2). **g,** Normalized peak areas of 3-phosphoserine and serine from wild-type or *ΔPHGDH* A375 cells from two independent knockout clones (#1, #2)) that were treated with vehicle (Veh, DMSO) or MTX (1 µM) for 15 h. **h,** PHGDH knockout A375 cells are auxotrophic for serine. The cell number of wild-type or *ΔPHGDH* A375 cells cultured in 10% dialyzed serum in the presence or absence of exogenous serine (400 µM) was measured over 5 days. **i,** Quantification of relative cell migration (transwell assay, 12 h) of A375 cells after treatment with the indicated doses of serine in serine/glycine-free growth media. **j,** Quantification of relative cell migration (transwell assay, 12 h) of A375 cells after treatment with the indicated doses of formate in growth media. **k,** Quantification of relative cell migration (transwell assay, 12 h) of A375 cells after vehicle (Veh) or MTX treatment (100 nM) in the presence or absence of SHMT1/2 inhibitor (SHIN1, 5 µM). **l,** Relative cell viability (Trypan blue) as in (a), but from A375 cells treated with vehicle (Veh, DMSO) or MTX (200 nM) in the presence or absence of SHMT1/2 inhibitor (SHIN1, 5 µM) for 48 h. **m,** Relative cell viability (Trypan blue) as in (a) but from A375 cells transfected with nontargeting controls (siCtl) or siRNA targeting MTHFD1L and treated with vehicle (Veh, DMSO) or MTX (200 nM) for 48 h. (a-d, g-m) Data are presented as the mean ± s.d from n=3 (a,b,g,h,l,m) or n=5 (c,d,i-k) of biologically independent samples. (a-d, g-m) One-way ANOVA, Turkey's post-hoc test, multiple comparison, *p<0.05, ***p<0.001, ****p<0.0001. Data are representative of n=2 (h,m) or n=3 (a-g,i-l) independent experiments. Source data and exact p values are provided as a Source Data file.

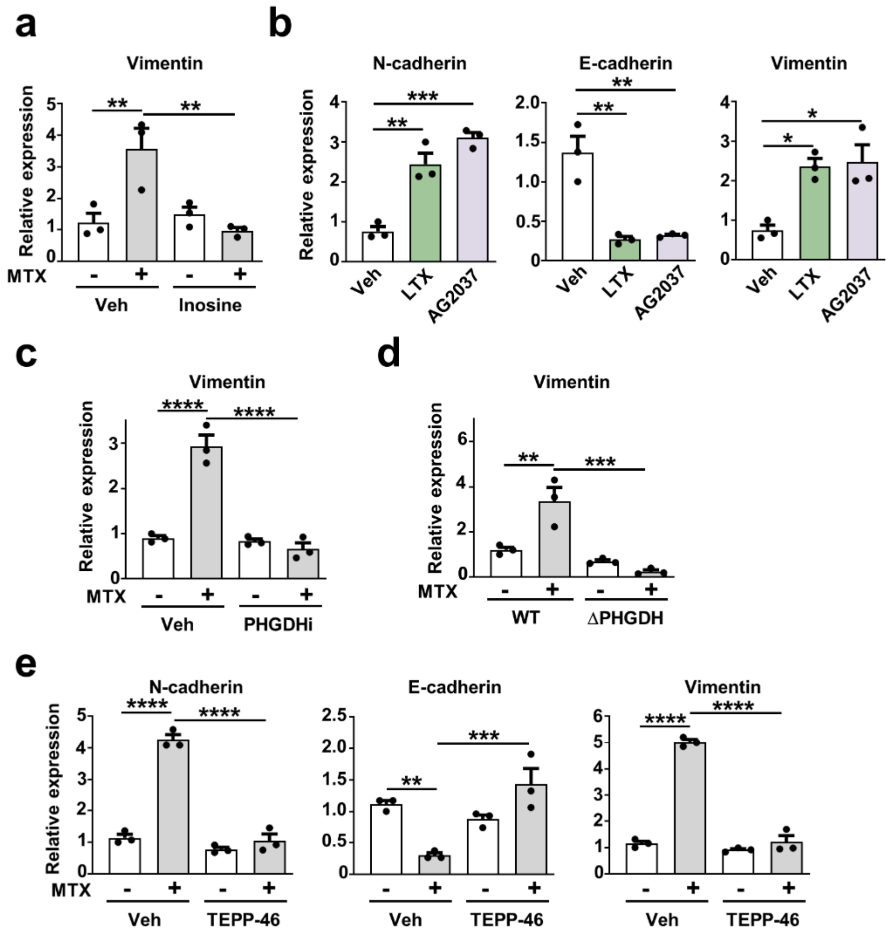

Supplementary Figure 6. Purine depletion initiates an epithelial-mesenchymal transition transcriptional program.

a, Relative mRNA expression of the indicated EMT marker Vimentin upon treatment of A375 cells with MTX (200 nM, 24 h) in the presence or absence of inosine (50 µM, 24 h). In support of Fig. 5c. **b,** Relative mRNA expression of the indicated EMT markers upon from A375 cells that were treated with DMSO (Veh), LTX (800 nM, 24 h), or AG2037 (100 nM, 24 h). **c,** Relative mRNA expression of Vimentin upon treatment of A375 cells with MTX (200 nM) in the presence or absence of PHGDH inhibitor (PHGDHi, BI-4924, 15 µM, 24 h). In support of Fig. 5e. **d,** Relative mRNA expression of the indicated EMT marker Vimentin from wild-type or *ΔPHGDH* A375 cells treated that were treated either vehicle (DMSO) or MTX (200 nM, 24 h). **e,** Relative mRNA expression of the indicated EMT markers from A375 cells that were treated with DMSO (Veh) or MTX (200 nM, 24 h) in the presence or absence of TEPP-46 (100 µM, 24 h). (a-e) Data are presented as the mean ± s.d from n=3 (a-e) of biologically independent samples. (a-e) One-way ANOVA, Turkey's post-hoc test, multiple comparison, *$p<0.05$, **$p<0.01$, ***$p<0.001$, ****$p<0.0001$. Data are representative of n=2 (a-e) independent experiments. Source data and exact p values are provided as a Source Data file.
