# Supplementary material for: Purine nucleotide depletion prompts cell migration by stimulating the serine synthesis pathway
Source: Nat Commun. 2022 May 16;13:2698. doi: 10.1038/s41467-022-30362-z (PMC9110385; doi:10.1038/s41467-022-30362-z)
Supplement: Supplementary file 3 — Reporting Summary [file 41467_2022_30362_MOESM3_ESM.pdf]

nature portfolio

Corresponding author(s): Gerta Hoxhaj, Issam Ben-Sahra, Brendan Manning

Last updated by author(s): Apr 13, 2022

Reporting Summary

Nature Portfolio wishes to improve the reproducibility of the work that we publish. This form provides structure for consistency and transparency in reporting. For further information on Nature Portfolio policies, see our Editorial Policies and the Editorial Policy Checklist.

Statistics

For all statistical analyses, confirm that the following items are present in the figure legend, table legend, main text, or Methods section.

n/a	Confirmed	
☐	☒	The exact sample size (*n*) for each experimental group/condition, given as a discrete number and unit of measurement
☐	☒	A statement on whether measurements were taken from distinct samples or whether the same sample was measured repeatedly
☐	☒	The statistical test(s) used AND whether they are one- or two-sided<br>*Only common tests should be described solely by name; describe more complex techniques in the Methods section.*
☒	☐	A description of all covariates tested
☒	☐	A description of any assumptions or corrections, such as tests of normality and adjustment for multiple comparisons
☐	☒	A full description of the statistical parameters including central tendency (e.g. means) or other basic estimates (e.g. regression coefficient) AND variation (e.g. standard deviation) or associated estimates of uncertainty (e.g. confidence intervals)
☐	☒	For null hypothesis testing, the test statistic (e.g. *F*, *t*, *r*) with confidence intervals, effect sizes, degrees of freedom and *P* value noted<br>*Give P values as exact values whenever suitable.*
☒	☐	For Bayesian analysis, information on the choice of priors and Markov chain Monte Carlo settings
☒	☐	For hierarchical and complex designs, identification of the appropriate level for tests and full reporting of outcomes
☒	☐	Estimates of effect sizes (e.g. Cohen's *d*, Pearson's *r*), indicating how they were calculated

Our web collection on statistics for biologists contains articles on many of the points above.

Software and code

Policy information about availability of computer code

Data collection	Mass spectrometry was performed with AB QTRAP 5500 (Applied Biosystems SCIEX);Thermo Scientific (Bremen, Germany) Q-Exactive HF-X hybrid quadrupole orbitrap high resolution mass spectrometer (HRMS), Seahorse XFe96 Analyzer (Seahorse BioScience), SpectraMax iD3 Multimode Plate Detection Platform (Molecular Devices, LLC) for absorbance, luminesence and fluorescence assay, Perkin-Elmer Enspire Multimode plate reader, Celigo image cytometer-4 Channel (Nexcelom Bioscience,UK), Primovert ZEISS microscope, LUNA-II™ Automated Cell Counter, Zeiss LSM 780 Laser Scanning Microscope, NanoZoomer 2.0-HT, slide scanner Zeiss Axioscan.Z1, BioRad CFX384 Touch Real-Time PCR Detection System, Benchmark Bead-Blaster Homogenizer (product code: D2400-R) from Marshall scientific,TraceFinder 5.1 (ThermoScientific). Images of mice for Figure 2f were obtained from Servier Medical Art (https://smart.servier.com) under a Creative Commons Attribution 3.0 Unported License(https://creativecommons.org/licenses/by/3.0/). The mice in the images in Figures 6a, 6h were obtained from Biorender (https://biorender.com/). The image in Figure 4a was assembled in Powerpoint, Microsoft 365®.
Data analysis	Graph Pad Prism™ 8.4.3 , Wave software (Agilent Technologies, Version 2.6.1 for Windows), TraceFinder 5.1 (ThermoFisher Scientific), MultiQuant 2.2.2 (Agilent Technologies, CA, version 2.1.1742.0), Adobe Fireworks 8®, Microsoft Excel 365®, Microsoft Powerpoint 365®, Celigo 5.1.0, ImageJ Java 1.8.0_172, ZEN 3.3.890000 (Blue ed) software, SoftMax Pro7 (version 7.1.0), MetaboAnalyst5.0 (www.metaboanalyst.ca),NDP.view 2 software (U12388-0),Bio-Rad CFX Manager version 3.1.1517.0823.

For manuscripts utilizing custom algorithms or software that are central to the research but not yet described in published literature, software must be made available to editors and reviewers. We strongly encourage code deposition in a community repository (e.g. GitHub). See the Nature Portfolio guidelines for submitting code & software for further information.

Data

Policy information about availability of data

All manuscripts must include a data availability statement. This statement should provide the following information, where applicable:

- Accession codes, unique identifiers, or web links for publicly available datasets
- A description of any restrictions on data availability
- For clinical datasets or third party data, please ensure that the statement adheres to our policy

Source data are provided with this paper. The data supporting our findings are available in the article, the supplementary files, or from the corresponding authors (B.D.M., I. B-S., or G.H.) upon request.

Field-specific reporting

Please select the one below that is the best fit for your research. If you are not sure, read the appropriate sections before making your selection.

☒ Life sciences ☐ Behavioural & social sciences ☐ Ecological, evolutionary & environmental sciences

For a reference copy of the document with all sections, see nature.com/documents/nr-reporting-summary-flat.pdf

Life sciences study design

All studies must disclose on these points even when the disclosure is negative.

Sample size	To ensure an adequate power of the statistical testing, we perform special analyses prior to running the experiments, to calculate how large an n is required. The null hypothesis (H0) = no difference between the untreated versus treated, while the alternative hypothesis (H1) = outcome where there is a difference. The power calculation is: power = P (reject Ho | H1 is true) = P (accept H1 | H1 is true). We estimate that the probability to obtain a difference between the groups is ~95% and that there is only 5% H0 is true. n = logβ/logp, where β is the probability of committing a Type II error (0.05) and p is the proportion of the animals in the group that are not affected by the treatments. To obtain at least 50% of mice altered by the treatment in one group, we will need: n=log(0.05)/log(0.5) = 4.32. Five animals were included in each experiment.
Data exclusions	Data were not excluded.
Replication	Each experiment in the figures was independently repeated successfully more than two-three times and similar results were obtained. Two biologically complementary in vivo metastasis experiments were performed.
Randomization	The assignment of the experimental conditions of the mice was randomized. The running of samples for mass spectrometry was also randomized. Microscopic images were taken randomly. Randomization was not included for experiments involving Western blot, pPCR, proliferation or migration assays. Imbalance of covariates is not relevant to these experiments. Appropriate statistical analysis was consistently performed to ensure significance or non-significance of the data with or without randomization.
Blinding	The in vivo experiment was blinded, as one person prepared and assigned numbers to mice and samples, and the other person performed injection without prior knowledge of the sample identity. Investigators were not blinded for in vitro experiments, such as western blotting, qPCRs, proliferation, or cell migration assays, but all the data was collected and analyzed without bias. The in vitro experiments showed results that could not be influenced by conscious or unconscious selection of statistical tests and reporting.

Behavioural & social sciences study design

All studies must disclose on these points even when the disclosure is negative.

Study description	N/A
Research sample	N/A
Sampling strategy	N/A
Data collection	N/A
Timing	N/A
Data exclusions	N/A
Non-participation	N/A

Randomization	N/A

Ecological, evolutionary & environmental sciences study design

All studies must disclose on these points even when the disclosure is negative.

Study description	N/A
Research sample	N/A
Sampling strategy	N/A
Data collection	N/A
Timing and spatial scale	N/A
Data exclusions	N/A
Reproducibility	N/A
Randomization	N/A
Blinding	N/A

Did the study involve field work? ☐ Yes ☒ No

Reporting for specific materials, systems and methods

We require information from authors about some types of materials, experimental systems and methods used in many studies. Here, indicate whether each material, system or method listed is relevant to your study. If you are not sure if a list item applies to your research, read the appropriate section before selecting a response.

Materials & experimental systems

n/a	Involved in the study	
☐	☒	Antibodies
☐	☒	Eukaryotic cell lines
☒	☐	Palaeontology and archaeology
☐	☒	Animals and other organisms
☒	☐	Human research participants
☒	☐	Clinical data
☒	☐	Dual use research of concern

Methods

n/a	Involved in the study	
☒	☐	ChIP-seq
☒	☐	Flow cytometry
☒	☐	MRI-based neuroimaging

Antibodies

Antibodies used

The following antibodies have been used in this study:

Alexa Fluor® 488, Cat. #8878, Cell Signaling Technology, 1:200, IF

Anti-S100, Cat. #Z0311, polyclonal, LOT:00060051, Dako, 1:500, IF

GART, Cat. #13659-1-AP, Proteintech, 1:1000, WB

p-S6 Kinase -T389, Cat. #9234, Lot:12, Cell Signaling Technology, 1:1000, WB

S6 Kinase, CST #2708, Lot:8, Cell Signaling Technology, 1:1000, WB

DHODH, Cat. #14877-1-AP, Proteintech, 1:1000, WB

APRT, Cat. #ab196558, Abcam, 1:1000, WB

HPRT, Cat. #sc-393901, Santa Cruz, 1:1000, WB

PKM1, Cat. #SAB4200094, LOT:117M4754V, Sigma, 1:1000, WB

PKM2, Cat. #4053S, LOT:6, Cell Signaling, 1:1000, WB

PHGDH, Cat. #14719-1-AP, Proteintech, 1:1000, WB

PSAT1, Cat. #10501-1-AP, Proteintech, 1:1000, WB

MTHFD1L, Cat. #16113-1-AP, Proteintech, 1:1000, WB

β-actin, Cat. #A5316, Sigma, 1:5000, WB

HRP-conjugated anti-mouse, Cat. #7076, Cell Signaling Technology, secondary antibody, 1:5000, WB

Anti-rabbit, Cat. #7074, Cell Signaling Technology, secondary antibody, 1:5000, WB

Validation

All antibodies were obtained from commercial vendors and the validity can be retrieved from the manufacturer's website.
We further made sure to identify the correct molecular weight for each of the antibodies.

Alexa Fluor® 488 (Cat. #8878, Cell signaling technology). This antibody can stain the cytoskeleton through the binding of phalloidin to F-actin. https://www.cellsignal.com/products/buffers-dyes/alexa-fluor-488-phalloidin/8878?Ns=product.sortId%7C0&N=102284+4294956287&fromPage=plp

Anti-S100 (Cat. #Z0311, Dako). https://www.agilent.com/en/product/immunohistochemistry/antibodies-controls/primary-antibodies/s100-(dakoomnis)-76198

GART (Cat. #13659-1-AP, Proteintech). https://www.ptglab.com/products/GART-Antibody-13659-1-AP.html

p-S6 Kinase -T389 (Cat. #9234, Cell Signaling Technology). https://www.cellsignal.com/products/primary-antibodies/phospho-p70-s6-kinase-thr389-108d2-rabbit-mab/9234

S6 Kinase (CST #2708, Cell Signaling Technology). https://www.cellsignal.com/products/primary-antibodies/p70-s6-kinase-49d7-rabbit-mab/2708

DHODH (Cat. #14877-1-AP, Proteintech). https://www.ptglab.com/products/DHODH-Antibody-14877-1-AP.html

APRT (Cat. #ab196558, Abcam). https://www.abcam.com/aprt-antibody-ab196558.html

HPRT (Cat. #sc-393901, Santa Cruz). https://www.scbt.com/p/hprt-antibody-b-11?requestFrom=search

PKM1 (Cat. #SAB4200094, Sigma). https://www.sigmaaldrich.com/US/en/product/sigma/sab4300663?gclid=Cj0KCQiAmpyRBhC-ARIsABs2EAqLC76TPAWEqHTEYF8tdP07cMCbh5g2qXu5LXIw94h6dRdTPV6ZoP8aAtGtEALw_wcB

PKM2 (Cat. #4053S,Cell Signaling). https://www.cellsignal.com/products/primary-antibodies/pkm2-d78a4-xp-rabbit-mab/4053

PHGDH (Cat. #14719-1-AP, Proteintech). https://www.ptglab.com/products/PHGDH-Antibody-14719-1-AP.html

PSAT1 (Cat. #10501-1-AP, Proteintech). https://www.ptglab.com/products/PSAT1-Antibody-10501-1-AP.html

MTHFD1L (Cat. #16113-1-AP, Proteintech). https://www.ptglab.com/products/MTHFD1L-Antibody-16113-1-AP.html

β-actin (Cat. #A5316, Sigma). https://www.sigmaaldrich.com/US/en/product/sigma/a5316?gclid=Cj0KCQiAmpyRBhC-ARIsABs2EAqJ7v2fSBrs90R0Mt7zAAXkzULtD8SvAXo-C_hVKyKnnHpRbpb0-TIaAk77EALw_wcB

HRP-conjugated anti-mouse (Cat. #7076, Cell Signaling Technology). https://www.cellsignal.com/products/secondary-antibodies/anti-mouse-igg-hrp-linked-antibody/7076

Anti-rabbit, Cat. #7074, Cell Signaling Technology). https://www.cellsignal.com/products/secondary-antibodies/anti-rabbit-igg-hrp-linked-antibody/7074

Eukaryotic cell lines

Policy information about cell lines

Cell line source(s)

HeLa, SK-MEL-28, A375, B16, CAL-51, A549, CCL-1, CCL-1.4 and LNCaP were from ATCC.

Authentication

Common cancer cells were authenticated via ATCC via short-tandem repeat profiling.

Mycoplasma contamination	All cell lines tested negative for mycoplasma.
Commonly misidentified lines (See ICLAC register)	No commonly misidentified cell lines were used.

Palaeontology and Archaeology

Specimen provenance	N/A
Specimen deposition	N/A
Dating methods	N/A

☐ Tick this box to confirm that the raw and calibrated dates are available in the paper or in Supplementary Information.

Ethics oversight	N/A

Note that full information on the approval of the study protocol must also be provided in the manuscript.

Animals and other organisms

Policy information about studies involving animals; ARRIVE guidelines recommended for reporting animal research

Laboratory animals	8 weeks old female athymic nude mice (002019, Jackson laboratory). Six-week-old female BALB/cJ (Jackson Laboratory) were also used. All procedures were approved by the Institutional Animal Care and Use Committee (IACUC) at University of Texas Southwestern Medical Center and at Northwestern University. The Animal Resource Center (ARC) at both institutions are fully accredited by the American Association for Accreditation of Laboratory Animal Care (AAALAC), and the animal care and use program conforms to standards in "The Guide for the Care and Use of Laboratory Animals," DHEW publication number (NIH) 78-23. The staff of ARC provides husbandry services under the guidance of supervisors who are certified as Animal Technologists by the American Association for Laboratory Animal Science (AALAS). Veterinary care is provided by ARC faculty members and veterinary technicians. All mice were housed in a pathogen free environment with a 12:12 light/dark cycle and fed chow diet ad libitum. The temperature in the animal facility is kept at 72F, with a range from 68-79F, humidity is kept at 33%, with a range between 30%-70%.
Wild animals	The study did not involved wild animals.
Field-collected samples	The study did not involve field-collected samples.
Ethics oversight	Experiments were conducted according to the protocols approved by Institutional Animal Care and Use Committee (IACUC) at University of Texas Southwestern Medical Center (Protocol # 2020-102880).

Note that full information on the approval of the study protocol must also be provided in the manuscript.

Human research participants

Policy information about studies involving human research participants

Population characteristics	N/A
Recruitment	N/A
Ethics oversight	N/A

Note that full information on the approval of the study protocol must also be provided in the manuscript.

Clinical data

Policy information about clinical studies
All manuscripts should comply with the ICMJEguidelines for publication of clinical research and a completedCONSORT checklist must be included with all submissions.

Clinical trial registration	N/A
Study protocol	N/A
Data collection	N/A
Outcomes	N/A

Dual use research of concern

Policy information about dual use research of concern

Hazards

Could the accidental, deliberate or reckless misuse of agents or technologies generated in the work, or the application of information presented in the manuscript, pose a threat to:

No	Yes	
x		Public health
x		National security
x		Crops and/or livestock
x		Ecosystems
x		Any other significant area

Experiments of concern

Does the work involve any of these experiments of concern:

No	Yes	
x		Demonstrate how to render a vaccine ineffective
x		Confer resistance to therapeutically useful antibiotics or antiviral agents
x		Enhance the virulence of a pathogen or render a nonpathogen virulent
x		Increase transmissibility of a pathogen
x		Alter the host range of a pathogen
x		Enable evasion of diagnostic/detection modalities
x		Enable the weaponization of a biological agent or toxin
x		Any other potentially harmful combination of experiments and agents

ChIP-seq

Data deposition

- [ ] Confirm that both raw and final processed data have been deposited in a public database such as GEO.
- [ ] Confirm that you have deposited or provided access to graph files (e.g. BED files) for the called peaks.

Data access links<br>*May remain private before publication.*	N/A
Files in database submission	N/A
Genome browser session<br>(e.g. UCSC)	N/A

Methodology

Replicates	N/A
Sequencing depth	N/A
Antibodies	N/A
Peak calling parameters	N/A
Data quality	N/A
Software	N/A

Flow Cytometry

Plots

Confirm that:

- [ ] The axis labels state the marker and fluorochrome used (e.g. CD4-FITC).
- [ ] The axis scales are clearly visible. Include numbers along axes only for bottom left plot of group (a 'group' is an analysis of identical markers).
- [ ] All plots are contour plots with outliers or pseudocolor plots.
- [ ] A numerical value for number of cells or percentage (with statistics) is provided.

Methodology

Sample preparation	N/A
Instrument	N/A
Software	N/A
Cell population abundance	N/A
Gating strategy	N/A

- [ ] Tick this box to confirm that a figure exemplifying the gating strategy is provided in the Supplementary Information.

Magnetic resonance imaging

Experimental design

Design type	N/A
Design specifications	N/A
Behavioral performance measures	N/A

Acquisition

Imaging type(s)	N/A
Field strength	N/A
Sequence & imaging parameters	N/A
Area of acquisition	N/A

Diffusion MRI ☐ Used ☐ Not used

Preprocessing

Preprocessing software	N/A
Normalization	N/A
Normalization template	N/A
Noise and artifact removal	N/A
Volume censoring	N/A

Statistical modeling & inference

Model type and settings	N/A
Effect(s) tested	N/A

Specify type of analysis: ☐ Whole brain ☐ ROI-based ☐ Both

Statistic type for inference (See Eklund et al. 2016)	N/A
Correction	N/A

Models & analysis

n/a	Involved in the study
☒	☐ Functional and/or effective connectivity
☒	☐ Graph analysis
☒	☐ Multivariate modeling or predictive analysis
